# The COVID-19 endemic in Vietnam: Contextual considerations and implications

**DOI:** 10.3389/fpubh.2023.997635

**Published:** 2023-03-13

**Authors:** Linh Phuong Doan, Minh Ngoc Le Vu, Giang Thu Vu, Huong Thi Le, Long Hoang Nguyen, Carl A. Latkin, Roger C. M. Ho

**Affiliations:** ^1^Institute for Global Health Innovations, Duy Tan University, Da Nang, Vietnam; ^2^Faculty of Medicine, Duy Tan University, Da Nang, Vietnam; ^3^Institute of Health Economics and Technology, Hanoi, Vietnam; ^4^Institute for Preventive Medicine and Public Health, Hanoi Medical University, Hanoi, Vietnam; ^5^Department of Global Public Health, Karolinska Institutet, Stockholm, Sweden; ^6^Bloomberge School of Public Health, Johns Hopkins University, Baltimore, MD, United States; ^7^Institute for Health Innovation and Technology (iHealthtech), National University of Singapore, Singapore, Singapore; ^8^Department of Psychological Medicine, Yong Loo Lin School of Medicine, National University of Singapore, Singapore, Singapore

**Keywords:** COVID-19, endemic, exit strategy, policy response, pandemic measures

## 1. Introduction

After successful containment of COVID-19 in 2020, Vietnam suffered high death tolls due to the escalated spread and severity of SARS-CoV-2 Delta variant (1). However, as soon as COVID-19 vaccine became available, the country quickly rolled out a vaccination program at an unprecedented scale and speed, having 100% two doses coverage for adults and 96.3% for children between 12 and 17 years old by April 2022 (2). Since November 2021, the novel SARS-CoV-2 Omicron variant has replaced the Delta variant to become the most common disease variant worldwide, characterized by lower severity and mortality, especially for vaccinated patients (3, 4). Its characteristics and current socioeconomic context have shown to be highly adaptable to the three-tiered treatment model introduced by the Vietnam Ministry of Health since October 2021 as (1) mild symptoms can be easily home-treated and (2) self-quarantined patients provide great relief to then overloading hospitals. In Vietnam, current record of April 2022 indicated a 0.002% fatality rate per week compared to the 0.004% average worldwide (5). Attention and efforts have gradually been shifted to economic recovery as control measures are lifted. Hospitality businesses and international travel have recently been approved to re-open under strict hygiene supervision as per Official Dispatch No. 1265/BYT-DP. Instant economic relief is also provided through assistance to unemployed workers and loan offers to companies for employee salaries or zero-interest loans for businesses suspended for more than 3 months as per Resolution No. 11/NQ-CP about the socio-economic recovery and Decree No. 15/2022/ND-CP regulating tax exemption and reduction policies according to Resolution No. 43/2022/QH15.

The last week of April 2022 recorded an average of 7,417 new cases and 8 deaths per day, indicating Vietnam's strong post-peak period (6). In the health press conference on 13th April, the Ministry of Health has proposed two response strategies for two different scenarios based on WHO's pandemic guidelines. Ideally, given current predictable nature, COVID-19 will be classified as endemic and exit strategies will be implemented accordingly: opening all business, travel activities and gradually lifting mask mandates. On the other hand, if a new variant emerges, the effectiveness of current vaccines will be re-evaluated and pandemic response measures such as lockdowns, social restrictions, and testing requirements will again be strictly undertaken. Although the number of new cases had decreased sharply and the society as well as economic had been restored by the end of 2022 (in the last 30 days of the year, there were ~12,000 new cases and 11 mortalities (7), Vietnamese Government still directed to closely monitor the situation of COVID-19 along with the development of new strains, continue to encourage citizens to take personal protective measures and vigorously implement the booster shots vaccination campaign to prevent future pandemic outbreaks. Simultaneously, it is required to effectively control other emerging and reemerging infectious diseases, such as monkeypox, dengue fever, adenovirus outbreak, preventing overlapping epidemics (7). Even under WHO and CDC's specific guidelines for pandemic and endemic response (8, 9), choosing the appropriate policy change window for either scenario is difficult and with consequences. As global guidelines were documented for the general population, Vietnam should consult previous attempts by countries with similar contexts instead of merely adopting global recommendations. This opinion article discusses implications for both scenarios.

## 2. Exit strategy: Key factors

Not all exit strategies during the post-peak period have resulted in success. The full or partial lifting of pandemic restrictions as happened in Europe and the USA has been followed by new surge in infections, hospitalizations, and deaths (10). Therefore, if the exit strategy is to succeed in Vietnam, apart from strengthening citizen-government cooperation, officials need to pay close attention to three key factors: (1) vaccination for populations at the highest risks of COVID-19 including the elderly, citizens with underlying medical conditions and healthcare worker, (2) vaccine quality, and (3) health system capacity.

Firstly, the role of the national vaccination program should not be underestimated. However, it is not the rate of vaccination in the whole population that correlates with the number of new cases. China, whose vaccination rates are slightly higher than Vietnam's at 83.98%, has recently seen a fresh outbreak of COVID cases (11). Experts suspected that vaccination rate in elderly citizens may be one underlying reason for such lack of correlation, which seemed to be reflected in the case of Hongkong. Despite the remarkable rate of 83.2% vaccinated of all populations, only 57.88% of Hongkong's senior citizens have received 2nd dose of vaccine and only 25.1% have received a 3rd dose (12). The latest outbreak of Omicron in Hongkong recorded a total of 6,569 deaths and ~246 deaths per day at its peak. The low vaccination rate among elderly, combined with low levels of mass immunity, has turned Hongkong's Omicron attack into one of the most critical surges in the world. Meanwhile, other Asian countries with higher elderly vaccination coverage such as South Korea record a lower cumulative death toll per capita (0.08%) compared to Hongkong (1.28%). Previous failures of COVID-19 relaxation measures and vaccination programs in Europe, USA, and Hongkong remind Vietnam of the time-sensitivity of application and the importance of vaccination for the elderly, should the same exit strategy be adopted. In Vietnam, the elderly (aged 60 and above) made up 12.8% of the total population in 2021, equivalent to 12.6 million people who usually contract chronic diseases like diabetes, high blood pressure, or coronary artery disease (13). Therefore, vaccination efforts for the elderly should be prioritized to create herd immunity, being prepared for the country's reopening. In fact, the Government has drastically implemented vaccination campaigns for the elderly, including mobile and in-home COVID-19 vaccination, particularly in major cities with a high density of population such as Ho Chi Minh City, Hanoi, Da Nang, Quang Nam, etc (14–16). Additionally, to sustain immunity in this population, it is also essential to give the elderly priority to types of vaccines that have higher efficacy among the elderly such as Pfizer/BioNTech COVID-19 vaccine (17). Vietnam had surpassed the WHO-recommended COVID-19 vaccine coverage rate of 70% by the first quarter of 2022, reaching a level of about 100% (18, 19).

Alongside the elderly, healthcare workers are also the top priority group in Vietnam's nationwide vaccination campaign. Working on the front lines against the pandemic, healthcare workers have been one of the most affected groups. Hence, since the program's inception in 2021, Vietnam has allocated the first injections to the medical workforce. Healthcare workers are one of 16 priority groups for vaccination in the plan to deploy the largest vaccination campaign in history from July 2021 to April 2022, when the COVID-19 epidemic situation was the most serious (20). At this point, maintaining a high immunization rate against COVID-19 among healthcare professionals, which helps ensure the backbone of the health system, is critical for any scenario.

Vietnam has been paying attention to vaccination: while all measures have been gradually reduced, vaccination coverage is continuously expanded to all age groups. To date, a total of 213,061,726 doses of vaccine have been administered (21). However, questions about the quality discrepancy between kinds of vaccines are unsettled. In the US, the only two FDA-approved COVID-19 vaccines are the Pfizer and Moderna (22). Both have received extensive research and proven efficacy of above 94% during clinical trials (17, 23). Vietnam, on the other hand, has provided licenses for 8 kinds of vaccines: AstraZeneca, Gam-COVID-Vac (SputnikV), COVID-19 vaccine Janssen (Johnson & Johnson), Spikevax (COVID-19 vaccine Moderna), Comirnaty (Pfizer—BioNTech), Vero Cell (China National Biotec Group (CNBG)/Sinopharm), Hayat-Vax and Abdala. While the Pfizer/BioNTech COVID-19 vaccine records 95% efficacy on average (17), the Sinopharm Vero Cell vaccine is effective on only ~51% of recipients and records lower efficacy among older people (24). There is, thus, no ground to assume that the immunity of people vaccinated with three doses of Sinopharm remains as long or as strong as those vaccinated with three doses of Pfizer, and it is important that consideration of vaccine quality should be included in calculations for herd immunity and the timing of endemic COVID-19 in Vietnam. Given a pattern observed in previous major pandemics, COVID-19 will likely not be eradicated but instead will be controlled by regular vaccinations (25–27). COVID-19 booster shots become the focus of health, not only in Vietnam but around the world. The Ministry of Health of Vietnam has provided guidance on supplemental vaccination, which helps to increase access to the COVID-19 vaccine as well as improve coverage rate (28). Information on the efficacy and side effects of booster shots was provided fully and reliably. The decision to take a booster shot is influenced by personal preference as well as the effectiveness of the COVID-19 vaccine booster. More importantly, the Government of Vietnam is unlikely to be able to provide COVID-19 boosters with a policy of supporting money for COVID-19 vaccinations for people with current economic losses. A previous study by Tran et al. revealed that the highest willingness to pay when vaccines are >90% effective, 6–12 months immunity, minor side effects, negligible mortality, major drawbacks if not vaccinated, and the generated willingness to pay is VND 188,000 (US$ 8.02) (29). As a result, the Vietnamese government can offer strategies that are reasonable, effective, and well-received by the citizens.

Besides adjustments on a government organizational level, the capacity of health systems, specifically testing capacity during the exit period, should be strengthened. With the restrictions on social distancing dropped, schools opening and businesses operating at full force, constant testing and tracing remain vital. Even when the majority of the population has been vaccinated and will only experience mild symptoms if infected, a resurgence of COVID-19 remains a likely threat. Access to testing helps identify early clusters and determine the effectiveness of the exit strategy. One example of successful testing and tracing strategy is South Korea. Having sufficient vaccination coverage before embarking on the exit strategy, the key factor that sets South Korea out for success is its rapid and extensive testing throughout the phase. Since March 2022, South Korea has implemented its famous response strategy known as “test, trace, treat” and officially classified COVID-19 Omicron variant as flu-like (30, 31). As of April, South Korea's new COVID-19 cases fell below 100,000 for the first time in 7 weeks, predicting a halt of the omicron wave (32).

The final complication of endemic COVID-19 regards quality of life. It is true that the star factor in Vietnam's success is unity. The community involved in preventing the pandemic with the slogan “every citizen is a soldier” was a wise choice of the Vietnamese government (33). However, the high level of cooperation does not only come from the caution of citizens themselves but mostly from strict enforcement on the government's part. In addition, Vietnam's one-party political system and administrative management mechanism ensure that orders for COVID-19 prevention and control are implemented synchronously from central to grassroots levels (34). Famous measures adopted by Vietnam during its peak periods are hefty fines of 3,000,000 VND for unreported infections, 2,000,000 VND for violating the mask mandates, and up to 20,000,000 VND for neglecting social restrictions. In other words, direct financial hits have stimulated unity across Vietnamese management levels. However, this behavior pattern suggests that if Vietnam declares the end of COVID-19 at this point, there is no guarantee that people will continue to practice public health measures. Therefore, as social activities come back borderless and “maskless,” a division between health groups will very likely come into existence. Vulnerable groups such as the elderly, children or people unable to get vaccinated and people with underlying medical conditions would be hard to integrate and adapt to this situation as well as be limited from participating in soon-to-be normal activities, such as large social gatherings, regards to their health and safety. This hinders full recovery of the quality of life for the population as a whole. Thus, the key to preventing the spreading of the COVID-19 pandemic is the involvement of community and behavioral change.

## 3. Emergence of a new variant and the conventional pandemic response

With the current rate of international traveling globally, the appearance of a new mutation is not unlikely. Indeed, a new variant XE, known as the “recombinant” of Omicron BA.1 and BA.2 strains, has been discovered in the UK (35). Although extensive research has not been done on this new omicron strain, at present, its characteristics appear to be similar and milder in virulence despite increasing number of cases (36). Indeed, higher transmissibility and weaker infection levels are common dynamics for any viral pathogen—therefore, if a new strain of COVID-19 occurs in the future, it will most certainly be weaker in comparison to existing major strains. If such mutation is to put Vietnam back in the pandemic phase, the benefits and damages of pandemic measures should first be weighed out.

Vietnam's pandemic measures are not entirely the same as those in Western countries due to its socialist approach toward disease control. If the pandemic state is declared, typical socialist advocacies such as nationalization of private hospitals, low-cost COVID-19 hospitalization, and quarantine cost are likely to be adopted again. While these approaches have contributed to Vietnam's earlier success in 2020, the same result cannot be guaranteed considering the current economic state. After nearly 3 years of focusing on disease control, Vietnam's labor market has been suspending overall profit to provide economic relief for its citizens. In the first half of 2021, Vietnam's GDP growth rate fell to 5.64% compared to 2019 pre-pandemic growth rate of 6.77%. During the peak of the fourth outbreak of COVID-19 in August 2021, Vietnam's economy suffered from a negative 6% growth rate (37). Total foreign investment capital into Vietnam in 2020 has decreased by 25% compared to 2019, and the unemployment rate had increased by 2.4% compared to the first quarter of 2021 (38). Therefore, continuous closures will pose new burdens on an already overwhelmed economy. Economic and public health experts need to work closely to evaluate the extent to which Vietnam's economy can tolerate before implementing any measure that has potential socioeconomic damages.

COVID-19 has taken a bigger toll on global economic growth than any recent diseases have. In developing economies, growth is expected to decline from 6.3 to 4.4% in 2023, and for advanced economies, it is predicted to drop down to 2.3% in 2023 (39). By the end of 2021, the global debt-to-gross domestic product ratio remained 28 percentage points above pre-pandemic levels (40, 41). Understandably, a clear pattern in all proposals for COVID-19 to enter endemic state is the inclusion of economic and social recovery as their prime reasons. For past diseases, socioeconomic impact might not have been the primary concern due to short periods of lockdown and concentration on disease treatment (42, 43). However, calculated global economic and social suffering indicated that efforts should be immediately directed toward reversal of poverty and inequality setbacks, rather than solely on disease control measures. In soon-coming adjustments of pandemic response strategies, it is vital that a socioeconomic viewpoint be included.

## 4. Conclusion

Besides intensive vaccination coverage and analysis of virus trends, considerations should be directed to relieving economic burden and settling social issues. The unique characteristics of COVID-19 calls for a new time frame in pandemic response guidelines that considers socioeconomic impacts. Given the vaccines employed and vaccination rates throughout the country, Vietnamese officials should experiment with the first scenario by partial lifting of restrictions, evaluating social responses and developing new steps accordingly. At a probable end of the 3-year fight against COVID-19, Vietnam needs to be extremely careful in choosing its approach for either scenario.

## Author contributions

LD and ML wrote the original draft and edited the manuscript. HL and LD conceptualized and reviewed original draft. GV, LN, LD, CL, and RH reviewed the original draft. LD reviewed and edited the final manuscript. All authors contributed to the article and approved the submitted version.
